# Efficacy and safety of combination therapy with vildagliptin and metformin versus metformin up-titration in Chinese patients with type 2 diabetes mellitus: study design and rationale of the vision study

**DOI:** 10.1186/1475-2840-12-118

**Published:** 2013-08-19

**Authors:** Li-Nong Ji, Chang-Yu Pan, Ju-Ming Lu, Hong Li, Qiang Li, Qi-Fu Li, Yong-De Peng, Hao-Ming Tian, Chen Yao, Zhi-Gang Zhao, Ru-Ya Zhang, Xiang-Ling Wang, Lei Wang

**Affiliations:** 1 Peking University People’s Hospital, 11 Xizhimen Nan Dajie, Xicheng District, Beijing, China; 2 Chinese PLA General Hospital, No. 28 Fuxing Street, Beijing 100853, China; 3 Sir Run Run Shaw Hospital, Zhejiang University Medical College, Hangzhou, China; 4 The 2nd Affiliated Hospital of Harbin Medical University, Harbin, China; 5 The First Affiliated Hospital of Chongqing Medical University, Chongqing, China; 6 Shanghai First People's Hospital, Shanghai, China; 7 West China Hospital, Sichuan University, Chengdu, China; 8 Peking University First Hospital, Beijing, China; 9 Henan Provincial People's Hospital, Zhengzhou, China; 10 Beijing Novartis Pharma Co. Ltd, Beijing, China

**Keywords:** VISION study, Vildagliptin, Type 2 diabetes, Study design, DPP-IV inhibitors

## Abstract

**Background and aim:**

Limitations of the currently recommended stepwise treatment pathway for type 2 diabetes mellitus (T2DM), especially the failure of monotherapies to maintain good glycemic control, have prompted use of early, more aggressive combination therapies.

The VISION study is designed to explore the efficacy and safety of vildagliptin as an add-on to metformin therapy compared with up-titration of metformin monotherapy in Chinese patients with T2DM.

**Methods:**

VISION, a 24-week, phase 4, prospective, randomized, multicenter, open-label, parallel-group study, will include 3312 Chinese T2DM patients aged ≥18 years who are inadequately controlled (6.5% >HbA1c ≤9%) by metformin (750–1000 mg/day). Eligible patients will be randomized to receive either vildagliptin plus metformin or up-titration of metformin monotherapy (5:1). Patients will also be subgrouped (1:1:1:1) based on their age and body mass index (BMI): <60 years and <24 kg/m^2^; <60 years and ≥24 kg/m^2^; ≥60 years and <24 kg/m^2^; and ≥60 years and ≥24 kg/m^2^.

**Conclusion:**

The VISION study will test the hypothesis that early use of combination therapy with vildagliptin and metformin will provide good glycemic control and will be better tolerated than up-titration of metformin monotherapy. The study will also correlate these benefits with age and BMI.

## Background

Type 2 diabetes mellitus, a chronic metabolic disorder of complex pathophysiology, is prevalent worldwide [1]. According to the 2011 estimates of the World Health Organization (WHO), there are currently approximately 346 million adults affected by diabetes globally [2]. In China, a total diabetes prevalence of 9.7% (92.4 million adults) was reported by the China National Diabetes and Metabolic Disorders Study in 2007–2008, while the prevalence of prediabetes was estimated to be 15.5% (148 million adults) [3]. The prevalence of diabetes was highest among individuals aged ≥60 years and among those with a body mass index (BMI) >30 kg/m^2^[3]. Management of diabetes aims at improving glycemic control, which is typically measured as reductions in glycated hemoglobin (HbA1c) [4]. Current guidelines define the target for glycemic control as HbA1c <7.0% or <6.5% [1,4]. Several classes of antihyperglycemic agents with different mechanisms of action are currently available. Metformin and the thiazolidinediones suppress insulin resistance while α-glucosidase inhibitors act within the gastrointestinal (GI) tract to lower postprandial glucose excursions. Sulfonylureas and meglitinides increase β cell insulin secretion in a glucose-independent manner. Dipeptidyl peptidase IV (DPP-IV) inhibitors and glucagon like peptide-1 (GLP-1) analogs improve insulin secretion and suppress glucagon secretion by glucose-dependent mechanisms [5].

Current clinical practice guidelines recommend a stepwise treatment pathway for diabetes [1]. Lifestyle modifications such as weight reduction, dietary adjustments, and physical exercise form the first step of treatment followed by initiation of monotherapy. Metformin is recommended as the first-line oral antihyperglycemic agent in most patients with T2DM by almost all clinical practice guidelines. Along with its favorable effect on blood glucose, metformin is associated with a low incidence of weight gain and hypoglycemia, and it also has cardioprotective properties [1,5-10]. Subsequent stepwise intensification of metformin monotherapy is recommended if glycemic control is inadequate [1,5,11]. In patients with persistent hyperglycemia even after the maximum effective and/or tolerated dose of metformin is used, the rapid addition of other antihyperglycemic agents is recommended [11]. If glucose control remains inadequate, another medication should be added to the initial therapy [1,11].

The stepwise treatment approach is, however, associated with several limitations. Lifestyle interventions are difficult to be implements and maintain, and have failed to achieve effective glycemic control alone [12]. Given the limitation of monotherapies to act on the multiple pathophysiological mechanisms involved in glucose control, they frequently fail to achieve the target glycemic goal [1]. Failure of monotherapies is also attributed to their inability to prevent deterioration of pancreatic β-cell function, which is commonly observed in diabetes [13,14]. Although monotherapies can provide initial glycemic control, several clinical studies have demonstrated failure of monotherapy to maintain long-term glycemic control [15]. The United Kingdom Prospective Diabetes Study (UKPDS 49), demonstrated that long-term monotherapy with either insulin, sulfonylureas, or metformin could not sustain the glycemic control initially achieved (HbA1c <7%). Approximately 50% and 75% of patients required the addition of at least one more pharmacological agent after 3 years and 9 years of follow-up, respectively [16]. A Diabetes Outcome Progression Trial (ADOPT) compared the durability of glycemic control in patients receiving rosiglitazone, metformin, or glyburide as initial monotherapy [17]. All these monotherapies eventually failed to sustain the targeted glycemic control over time. Furthermore, all were associated with high discontinuation rates, mainly due to drug-related adverse effects [17]. In another large observational study undertaken in the UK, approximately 50% of patients failed to achieve the target HbA1c level of <7% during the first year with sulfonylurea or metformin monotherapy. After 3 years of follow-up, neither of the monotherapies was able to maintain the target HbA1c in >70% patients who had initially achieved the goal during the first year [14]. Moreover, these agents were associated with several adverse events (AEs) such as hypoglycemia, weight gain, GI disturbances, peripheral edema, and potential cardiovascular effects [8].

As recommended in the traditional stepwise approach, intensification of therapy with higher doses has shown to be successful in improving glycemic control. However, the increased risk of AEs with dosage increases has resulted in impaired patient compliance and subsequently poor glycemic control [12]. For example, metformin therapy is associated with dose-dependent GI adverse effects such as diarrhea, flatulence, and abdominal discomfort, which often lead to treatment discontinuation [18,19]. In the UKPDS 33 study, intensive treatment with sulfonylureas or insulin was associated with an increased incidence of hypoglycemia and weight gain [20]. Moreover, the stepwise approach can also cause a delay in switching from monotherapy to combination therapy, which often leads to further deterioration of glycemic control [1,15,19].

Thus, limitations of the stepwise treatment approach and the progressive decline in pancreatic β-cell function in diabetes warrants new treatment strategies. Early use of more aggressive combination therapy, before responsiveness to monotherapy begins to decline, can be an effective approach [1,15]. This approach may provide several advantages, including greater glycemic control and the ability to act on different pathological mechanisms involved in glucose dysregulation [1]. Additionally, early interventions are particularly beneficial as they can slow the onset and progression of T2DM and its associated complications [1]. Combination therapy typically requires lower doses than those required for individual monotherapies, which can reduce the adverse effects associated with higher doses of monotherapy and thus improve tolerability [15]. The EMPIRE study demonstrated that early use of combination therapy comprising submaximal doses of antihyperglycemic agents can improve glycemic control without significantly increasing the occurrence of adverse effects. The use of rosiglitazone, a thiazolidinedione, as add-on therapy with submaximal doses of metformin was shown to be non-inferior to up-titration of metformin to its maximal effective dose in reducing HbA1c [19]. Furthermore, add-on therapy was better tolerated than metformin alone, with a lower incidence of GI AEs [19]. However, the potential cardiovascular effects associated with rosiglitazone has limited the clinical use of this combination therapy [1]. Thiazolidinediones are also associated with weight gain and thus the benefits of their combination with metformin should be weighed against the potential risk. Similarly, use of traditional combinations such as metformin with sulfonylureas, or metformin with insulin has limited clinical use owing to the associated hypoglycemia and weight gain [1,19,21]. Thus, the critical question that still remains to be answered is: which antihyperglycemic agents can be used effectively at early stages in the treatment of T2DM to effectively complement metformin? [13,21].

The multiple physiological actions of the incretin hormone glucagon-like peptide-1 (GLP-1), which include sensitization of β cells, augmentation of glucose-stimulated insulin secretion, inhibition of glucagon secretion, delayed gastric emptying, and stimulation of insulin biosynthesis make it a potentially useful therapeutic agent for the treatment of diabetes [22]. However, as GLP-1 is rapidly degraded by DPP-IV enzyme, which limits its effects, the use of DPP-IV inhibitors to prolong the half-life of the circulating endogenous peptide has proved an effective approach to developing new therapeutic antidiabetic agents [22]. Recent reviews of randomized studies of a minimum of 12 weeks’ duration have suggested that DPP-IV inhibitors are well tolerated, with no reports of severe hypoglycemia and weight gain in patients with T2DM [23,24].

Vildagliptin, a potent, selective, and orally active second-generation DPP-IV inhibitor, was recently approved for the treatment of T2DM. Vildagliptin has been shown to reduce fasting plasma glucose (FPG) and postprandial plasma glucose (PPG) levels and 24-hour glycemic excursions, and suppress postprandial plasma glucagon levels by inhibiting the activity of DPP-IV enzyme to raise the levels of fasting and postprandial active incretins [25-28]. It also improves insulin sensitivity, augments meal/postprandial plasma insulin levels, and enhances both α- and β-cell functions [28-31]. Improvements in functioning of the islets of Langerhans in individuals with well-controlled T2DM receiving vildagliptin under fasting conditions suggest a role beyond the enhancement of meal-induced GLP-1 and glucose-dependent insulinotropic polypeptide (GIP) activity [32]. In drug-naïve patients with mild hyperglycemia, vildagliptin reduced the progressive deterioration of glycemic control over 2 years. This indicates a favorable effect in preventing the deterioration of β-cell function [33]. In clinical studies, vildagliptin has been shown to improve glycemic control when administered as monotherapy or combination therapy in drug-naïve patients and in treatment-experienced patients [13,25]. In drug-naïve patients with T2DM, significant and progressive reductions in HbA1c were seen over 12 weeks of vildagliptin monotherapy (100 mg) [34].

The use of vildagliptin in combination with metformin has been reported to be well tolerated, with no reports of weight gain or severe hypoglycemia [35]. In patients poorly controlled with insulin monotherapy, the effectiveness of add-on vildagliptin therapy was investigated in a 24-week, double-blind, randomized clinical trial. The addition of vildagliptin not only improved glycemic control but also reduced the severe hypoglycemic events associated with insulin monotherapy [36]. Moreover, vildagliptin has also been found to have a weight-neutral effect, both as monotherapy and as combination therapy in patients with T2DM [25].

These reports have raised an important question – will a combination of vildagliptin plus metformin be both effective in achieving glucose control and well tolerated? The multiple mechanisms of action of vildagliptin in regulating glucose levels can complement the action of metformin, which lowers plasma glucose without affecting insulin secretion. Vildagliptin exerts its glucose-lowering effect through an increase in glucose-dependent insulin secretion, improving the sensitivity of both α and β cells to glucose, as well as suppressing FPG, PPG and postprandial glucagon secretion [13,25,26,37]. Several studies have demonstrated the superior efficacy of vildagliptin/metformin combination therapy in achieving target glycemic goals in comparison with metformin monotherapy [9,13,21,34]. The combination regimen was also safer, with no increased risk of hypoglycemia, weight gain or cardiovascular events [13]. Long-term vildagliptin/metformin combination therapy has also been shown to have beneficial effects on β-cell function [26]. Taken together these findings provide a rationale for the clinical use of vildagliptin plus metformin in patients with T2DM.

The VISION study ‒ VIldagliptin 50 mg bid as an add-on to metformin 500 mg bid compared with metformin up to 1000 mg bid in Chinese patients with type 2 diabeteS Inadequately controlled on metformin 500 mg bid mONotherapy ‒ was designed to explore differences in the efficacy and safety of vildagliptin as add-on therapy to metformin compared with up-titration of metformin in patients with T2DM inadequately controlled on metformin monotherapy. This study will therefore address the question as to whether the combination of submaximal metformin and vildagliptin will provide equivalent glucose control and less AEs compared with metformin up-titration. It also aims to evaluate if the beneficial effects of vildagliptin/metformin combination therapy can be correlated with various factors such as obesity and patient age. The efficacy and safety results of the VISION study will be published in due course after its completion.

## Materials and methods

### Subjects

Male and female Chinese T2DM patients (WHO/IDF criteria [38]) aged >18 years with HbA1c levels ranging between 6.5% and 9.0% and BMI between 22 and 45 kg/m^2^ at visit 1 who have received metformin at a stable dose of 750–1000 mg daily for at least 12 weeks before screening will be enrolled in the study. The patients will be required to maintain their individual eating and exercise habits during the study, and to follow all the study requirements. Written informed consent will be obtained from each patient prior to enrollment. Exclusion criteria for the study are listed in Table 1.

**Table 1:** Exclusion criteria for the VISION study

	
**1.**	Pregnant or lactating women
**2.**	Medical history of following diseases:
	• Type 1 diabetes mellitus or diabetes caused by pancreatic injury or secondary diabetes: Cushing syndrome or acromegaly
	• Acute complications of diabetes: ketoacidosis or non-ketotic hyperosmolar coma within the past 3 months
	• Acute infections within 4 weeks prior to the screening (visit 1) that may affect the efficacy and safety of the study
	• Any obvious diabetic complications such as symptomatic autonomic neuropathy, gastroparesis, worsening hyperglycemia in the absence of any comorbid illnesses, and conditions that may affect blood glucose
	• History of kidney disease or clinical diagnosis of renal insufficiency indicated by serum creatinine ≥132 μmol/L (≥1.5 mg/dL) in males, and ≥123 μmol/L (≥1.4 mg/dL) in females
	• History of a liver disease such as cirrhosis, hepatitis B, or hepatitis C (except carriers) or Alanine transaminase (ALT), aspartate aminotransferase (AST) greater than 2 times the ULN or total bilirubin greater than 2 times the ULN
	• History of acute and chronic pancreatitis
	• Malignant tumor in the past 5 years, including leukemia and lymphoma (except for carcinoma in situ of the skin)
	• Torsades de pointes ventricular tachycardia or persistent, clinically relevant ventricular tachycardia or ventricular fibrillation or second-degree atrioventricular block (Mobitz type I and II) or third-degree atrioventricular block, or QTc prolongation (>500 ms)
	• Myocardial infarction, coronary artery bypass surgery or percutaneous coronary intervention, unstable angina, or stroke within the past 6 months
	• Congestive heart failure requiring medical treatment
**3.**	Fasting plasma glucose >15 mmol/L (>270 mg/dL) at visit 1
**4.**	Clinically significant thyroid-stimulating hormone levels outside the normal range at visit 1
**5.**	Use of concomitant medications:
	• Other antihyperglycemic agents besides metformin within 12 weeks of visit 1
	• Long-term glucocorticoids (>7 consecutive days of treatment) within 4 weeks of visit 1
	• Treatment with growth hormone or similar drugs
	• Treatment with class Ia, Ib, or Ic, or class III antiarrhythmics
	• Treatment with any drug with known and frequent toxicity to a major organ system within the past 3 months
**6.**	Use of other investigational drugs at visit 1, or within 30 days or 5 half-lives of visit 1, whichever is longer
**7.**	History of active substance abuse (including alcohol) within the past 2 years
**8.**	Potentially unreliable patients or patients who, in the opinion of the investigator, are unsuitable for the study

ULN: upper limit of normal.

### Study design

**Figure 1:**
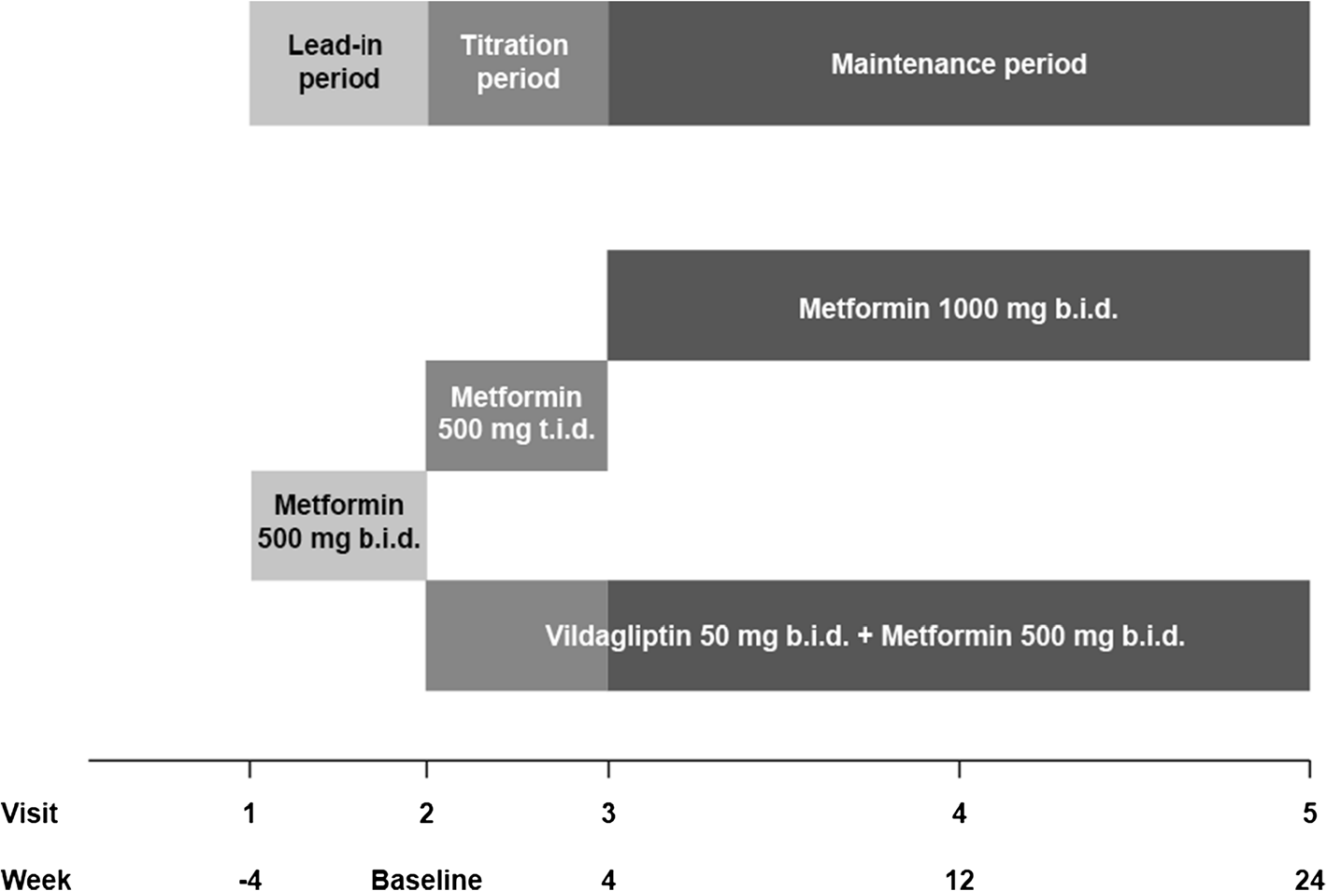
Design of the VISION study.

The VISION study is a 24-week prospective, randomized, multicenter, open-label, parallel-group controlled study. The planned study design is represented in Figure 1.

Patients participating in the study will be subgrouped (1:1:1:1) based on their BMI and age into 4 groups: (1) <60 years and BMI <24 kg/m^2^; (2) <60 years of age and BMI ≥24 kg/m^2^; (3) ≥60 years of age and BMI <24 kg/m^2^; and (4) ≥60 years of age and BMI ≥24 kg/m^2^. In each group, patients will be randomized according to the study design. At visit 1, patients meeting the inclusion criteria will be assigned to open-label metformin 500 mg bid for a 4-week lead-in period. At visit 2, patients will be randomized in a ratio of 5:1 to receive either vildagliptin 50 mg bid plus metformin 500 mg bid (Group A) or metformin 500 mg tid (Group B) for the next 4 weeks. From visit 3 to the end of the study, Group A patients will continue to receive the maintenance dose of vildagliptin 50 mg bid plus metformin 500 mg bid, while group B patients will receive metformin 1000 mg bid.

The primary, secondary, and exploratory objectives of the study are shown in Table 2. The study drug will be discontinued if it shows unsatisfactory efficacy at week 12 (i.e., FPG >13.3 mmol/L [240 mg/dL]) confirmed by a repeated measurement in the absence of any concurrent illness. Worsening of glucose levels in the absence of any other explainable concurrent disease or emergency condition that can affect glucose levels, the occurrence of AEs including GI side effects or clinically significant changes in laboratory parameters, or an abnormality that, in the opinion of the investigators, requires immediate withdrawal of treatment, will also lead to the study drug being discontinued. An inexplicable increase in levels of liver enzymes (aspartate transaminase, alanine aminotransferase and total bilirubin) without any clinical signs that is confirmed by a repeat measurement within 3 working days, pregnancy, severe or frequent hypoglycemia (i.e., unexplained hypoglycemic events requiring the assistance of another person or >3 hypoglycemic events within a week), treatment with prohibited concomitant medications, or any other deviation from the treatment protocol will also lead to treatment discontinuation. The reason for a patient discontinuing treatment will be recorded in the case report form.

**Table 2:** Primary, secondary, and exploratory objectives of the VISION study

	
**1.**	**Primary objective:**
	• To demonstrate that the change from baseline in HbA1c levels after 24 weeks of treatment with vildagliptin 50 mg bid as add-on therapy to metformin 500 mg bid is non-inferior to high-dose metformin
**2.**	**Secondary objectives:**
	• To demonstrate in predefined patient subgroups [based on body mass index (BMI <24 and ≥24 kg/m^2^) and age (<60 and ≥60 years)] that vildagliptin add-on therapy to metformin is non-inferior to high-dose metformin in the change from baseline in HbA1c levels
	• To determine the percentages of patients achieving the target HbA1c level of ≤6.5% in the two treatment arms of the overall population and in the predefined subgroups
	• To determine the percentages of patients achieving the target HbA1c level of ≤6.5% in the two treatment arms of the overall population and in the predefined subgroups
	• To determine the mean change from baseline to 24 weeks in fasting plasma glucose in the overall population and in the predefined subgroups
	• To determine the mean change from baseline to 24 weeks in 2-hour postprandial glucose in a subsample of 464 patients with type 2 diabetes mellitus
	• Safety analysis
**3.**	**Exploratory objectives:**
	• To determine the mean change from baseline to 24 weeks in body weight and lipid parameters in the overall population and in the predefined subgroups
	• To determine the change from baseline to 24 weeks in β-cell function in a subsample of 464 patients with type 2 diabetes mellitus

BMI: body mass index; HbA1c: glycated hemoglobin.

Treatment will also be discontinued if the patient cannot tolerate the GI symptoms associated with a dose increase at visit 2. If patients in Group B cannot tolerate the GI symptoms at visit 3, the dose of metformin can be decreased by one tablet (250 mg) until the GI symptoms improve. After that, the dose will be gradually increased over 1–2 weeks. The dose of the metformin in Group B will be at least 500 mg bid until week 12. After week 12 (visit 4), dose adjustments of metformin will not be allowed at any time. Adjustment of the vildagliptin dose will not be allowed throughout the study.

Patients discontinuing the study drug, including those who do not attend the last visit, will be assessed for safety within 30 days of receiving their last dose. If all randomized patients complete the 24-week, open treatment period, the study will be considered completed. Premature termination of the study will be notified to the institutional review boards and/or ethics committees.

### Study assessments

The primary efficacy assessment is the change in HbA1c levels from baseline. Secondary efficacy assessments include changes in FPG, the percentage of patients with HbA1c ≤6.5%, the percentage with HbA1c ≤6.5% without GI side effects, and changes in 2-hour PPG levels. Exploratory assessments include body weight, the serum lipid profile, and β-cell function.

Safety assessments include any AEs, serious AEs, unexplained elevations of liver enzymes, hypoglycemic events, physical examination findings, vital signs, height and weight, laboratory determinations (hematological and biochemical parameters, urine analysis), electrocardiogram, pregnancy and reproductive ability evaluation, appropriateness of the safety tests, and other indexes such as a standard meal test.

Patients will be assessed at visit 1 (week −4), visit 2 (baseline), visit 3 (week 4), visit 4 (week 12), and visit 5 (week 24). Patients will be instructed to fast overnight and not take the study drug before the visits at these times. The schedule of assessments is provided in Table 3. At visit 1, demographic information, the patient’s relevant medical history and history of diabetes and its complications (including the date when diabetes was confirmed; the presence or absence of proliferative retinopathy, non-proliferative retinopathy, nephropathy, neuropathy, or foot ulcers; and the dates these complications were confirmed) will be collected. Thyroid-stimulating hormone levels will also be evaluated at this visit. As there is no central laboratory for the study, all parameters will be tested locally.

**Table 3:** Schedule of assessments

**Visit**	**1**	**2**	**3**	**4**	**5**^**a**^
**Week**	**−4**	**Baseline**^**b**^	**4**	**12**	**24**
**Screening:**					
Informed consent	X				
Inclusion/exclusion criteria	X	X			
Height	X				
Thyroid-stimulating hormone	X				
**Demography/baseline data:**					
Demographic information	X				
History of diabetes and its complications	X				
History/current status	X	X			
**Treatment evaluation:**					
Drugs dispensed	X	X	X	X	
Drug count/check		X	X	X	X
Dosage records – metformin	X	X	X	X	X
Dosage records – vildagliptin		X	X	X	X
Currently receiving antihyperglycemic drugs	X				
Concomitant medicines	X	X	X	X	X
**Validity assessment:**					
HbA1c^c^	X	X		X	X
Fasting plasma glucose	X	X	X	X	X
Postprandial glucose^d^		X			X
Insulin^d^		X			X
Body weight	X	X	X	X	X
Standard meal test		X			X
**Safety evaluation:**					
Physical examinations	X	X			X
Vital signs	X	X	X	X	X
Electrocardiogram examination	X				X
Hematology examinations	X	X			X
Serum amylase		X	X	X	X
Standard biochemical tests^e,f^	X	X		X	X
Liver function tests^f^			X		
Pregnancy test^g^	X				X
Routine urine test		X			X
Adverse events	X	X	X	X	X
**Study end**					X

^a^Must also be taken in case of early withdrawal.

^b^Baseline =1st day of study.

^c^Determination of the HbA1c level with no central laboratory requires high-performance liquid chromatography.

^d^Only in the standard meal group.

^e^Standard biochemical examinations include, but are not limited to, the following: fasting blood glucose, liver function, kidney function (serum creatinine and blood urea nitrogen), and lipids (cholesterol and triglycerides).

^f^Liver function tests include ALT, AST, total and direct bilirubin, and alkaline phosphatase.

^g^Pregnancy tests specifically designed for women of childbearing potential.

HbA1c: glycated hemoglobin.

### Data analysis

Intention-to-treat (ITT) analysis will be performed. The full analysis set (FAS) will include all randomized patients who took the study drugs at least once and had at least 1 primary or secondary efficacy evaluation after baseline. For assessment of missing primary efficacy variables, the last observation carried forward (LOCF) technique will be used. The per-protocol set (PPS) will include ITT patients completing at least 22 weeks of treatment, and those who discontinued the study due to a poor therapeutic response (FPG >13.3 mmol/L [240 mg/dL]) after 12 weeks of treatment, provided they have no major protocol deviations and had a valid assessment of HbA1c levels within 7 days after their last dose of study drug. Any major deviation from the protocol will be identified and documented in the analysis plan. Patients will be analyzed in the treatment group to which they were randomly assigned for the efficacy assessment.

The safety analysis set (SAS) will include patients who took the study drugs at least once and had at least 1 safety assessment after baseline. The safety analyses will be according to the patients’ treatment, and will include the description of any AEs.

The FAS set will be used as the primary population for the primary efficacy variable, the change from baseline in HbA1c levels at 24 weeks. The primary efficacy variable will be analyzed using an analysis of covariance (ANCOVA) model and the LOCF approach.

Non-inferiority of the study drug will be analyzed using the null hypothesis and a unilateral alternative hypothesis, which are defined as follows:

$$H_{0},\delta_{\mathrm{vildagliptin}}\geq \delta_{\mathrm{metformin}}+0.3\%$$

$$H_{a},\delta_{\mathrm{vildagliptin}}<\delta_{\mathrm{metformin}}+0.3\%$$

where δ represents the change from baseline.

In order to control the overall type I error at alpha = 0.05, a sequential test procedure along with the Hochberg procedure will be employed. Non-inferiority testing for the overall population will be performed first. After achieving non-inferiority for the overall population, non-inferiority testing for the 4 subgroups will then be performed. Secondary efficacy variables include percentages of patients achieving therapeutic goals at the study endpoint: HbA1c ≤6.5%, HbA1c ≤6.5% without GI side effects, and absolute decreases in HbA1c from baseline of ≥1%, ≥0.7%, and ≥0.5%. In the FAS and PPS populations, the percentages of patients achieving the therapeutic goals will be analyzed and compared between treatment groups using the Chi-square test.

To assess the robustness of the results of the primary efficacy analysis, i.e., the change from baseline in HbA1c at 24 weeks, an ANCOVA model with LOCF will be used for the PPS. For the primary and secondary efficacy endpoints, subgroup analyses based on age and BMI will be performed.

Safety analyses will include summaries of AEs, changes from baseline in vital signs, the number of subjects with post-baseline laboratory values that will fall outside predetermined ranges, and the frequency and severity of hypoglycemic events. Adverse events will be summarized by system organ class and preferred terms. Summaries of AEs by severity and by relationship to study drug will also be provided.

The incidence of patients with any GI events will be compared between treatment groups using Fisher’s exact test.

### Sample size estimations

Non-inferiority testing will be performed on the subgroups of patients based on age and BMI. Assuming a treatment group difference of 0, standard deviation of 1, and a non-inferiority margin of 0.5, 690 patients will provide 90% power at a 1-sided with significance level of 0.05. Allowing for a 20% dropout rate, the number of subjects required for randomization is 690/ (1+20%) = 828 patients, yielding a total of 4 × 828 = 3312 subjects. For the estimation of the sample size, nQuery Advisor®, version 7.0, will be used.

### Ethics

The design, implementation, and reporting of the VISION study are strictly in compliance with the International Conference on Harmonization (ICH) of Technical Requirements for Registration of Pharmaceuticals for Human Use Guideline for Good Clinical Practice (GCP), and the Helsinki Declaration of ethical principles [39,40].

## Discussion

The inability of monotherapies to act on the multiple pathophysiological mechanisms involved in T2DM and to maintain good glycemic control as a result of progressive deterioration of β-cell function provide the rationale for the early use of combination therapy with different classes of drugs [1,13]. As metformin lowers plasma glucose levels without affecting insulin secretion, the addition of an agent such as vildagliptin which has a stimulatory action on insulin secretion is a suitable choice for combination therapy in patients with T2DM [13,25,37]. Metformin has shown an incremental effect on levels of GLP-1 in obese subjects without diabetes via mechanisms other than DPP-IV inhibition [41,42]. An additive effect on levels of intact GLP-1 has also been reported in patients with T2DM receiving metformin and vildagliptin concomitantly as compared with drug-naïve patients receiving only vildagliptin [13], which further supports the use of this combination of drugs [41,42].

The efficacy and safety of metformin and vildagliptin combination therapy has been evaluated in several placebo-controlled and active-controlled trials. The addition of vildagliptin to a stable dose of metformin monotherapy has been shown to be effective in sustaining glycemic control for at least 1 year, and in improving β-cell function and reducing insulin resistance and inflammatory markers [43-47]. A recent study of vildagliptin/low-dose metformin combination therapy in treatment-naïve patients with T2DM showed superior glycemic control and favorable GI tolerability compared with high-dose metformin therapy. This suggests the potential of vildagliptin/metformin combination therapy in the management of T2DM [21]. A recent phase III study in patients with inadequate glycemic control on low-dose metformin (500 mg bid) demonstrated that the addition of vildagliptin 100 mg qd to low-dose metformin 500 mg bid resulted in a larger reduction in HbA1c as compared with up-titration of metformin therapy to 1000 mg bid. Moreover, the combination therapy was well tolerated without any increase in hypoglycemic events, and fewer GI events as compared with high-dose metformin monotherapy. Thus, early and more aggressive therapy in T2DM is more beneficial and can be considered in patients with poor glycemic control with metformin monotherapy [9].

In comparison with Caucasians, Asians have higher body fat at lower BMI levels and are thus more prone to obesity and related disorders such as diabetes mellitus, dyslipidemia, and hypertension at a lower BMI [48]. Consequently, in China, the BMI cut-offs for ‘overweight’ (24 kg/m^2^) and ‘obesity’ (28 kg/m^2^) are lower than those of the WHO criteria, and the population aged 60 years or more is defined as ‘elderly’ [49]. The VISION study will include Chinese patients with inadequate glycemic control (HbA1c 6.5%-9.0%), despite being on metformin monotherapy. The study will categorize patients into 4 subgroups according to their age and BMI. Patients in each group will be randomized to receive vildagliptin (50 mg bid) plus metformin (500 mg bid) or metformin (1000 mg bid) in a ratio of 5:1. As both obesity and age are independent risk factors for the development of T2DM and also influence the efficacy of any antidiabetic therapy, the VISION study will evaluate the efficacy and safety of the vildagliptin/metformin combination according to age and BMI in comparison with high-dose metformin.

## Conclusion

The VISION study aims to prove the efficacy and safety of early intensive combination therapy with vildagliptin and low-dose metformin in comparison with gradual upward titration of metformin therapy in Chinese T2DM patients who are inadequately controlled on low-dose metformin monotherapy. This report describes the rationale for and the design of the VISION study, the efficacy and safety results of which will be published when the study is completed. The key design elements and results of the study will also be communicated to public databases such as ClinicalTrials.gov.

## Abbreviations

AE: Adverse event; ANCOVA: Analysis of covariance; BMI: Body mass index; DPP-IV: Dipeptidyl peptidase IV; FAS: Full analysis set; FPG: Fasting plasma glucose; GI: Gastrointestinal; GIP: Glucose-dependent insulinotropic polypeptide; GLP-1: Glucagon-like peptide-1; HbA1c: Glycated hemoglobin; IDF: International Diabetes Federation; ITT: Intention-to-treat; LOCF: Last observation carried forward; PPG: Postprandial plasma glucose; PPS: Per-protocol set; SAS: Safety analysis set; T2DM: Type 2 diabetes mellitus; UKPDS: United Kingdom Prospective Diabetes Study; WHO: World Health Organization.

## Competing interests

The authors declare that they have no competing interests.

## Authors’ contributions

LNJ, CYP, CY and RYZ conceived the study, participated in its design, and reviewed and edited the manuscript along with XLW and LW. JML, HL, QL, QFL, YDP, HMT, ZGZ participated the study design and reviewed the manuscript. All authors read and approved the final manuscript.
